# Promising early outcomes in surgical aortic valve replacement utilizing the rapid deployment approach for isolated aortic valve regurgitation

**DOI:** 10.1093/icvts/ivaf147

**Published:** 2025-06-24

**Authors:** Julia von der Linden, Polyxeni Vlachea, Olesya Kolos, Florian Herrmann, Sergey Belyaev, Gerd Juchem, Sven Peterss, Christian Hagl, Alexey Dashkevich

**Affiliations:** Department of Cardiac Surgery, Ludwig Maximilian University of Munich, Munich, Germany; Department of Cardiac Surgery, Ludwig Maximilian University of Munich, Munich, Germany; Department of Cardiac Surgery, Ludwig Maximilian University of Munich, Munich, Germany; Department of Cardiac Surgery, Ludwig Maximilian University of Munich, Munich, Germany; Munich Heart Alliance, Partner Site German Centre for Cardiovascular Disease (DZHK), Munich, Germany; Department of Cardiac Surgery, Ludwig Maximilian University of Munich, Munich, Germany; Department of Cardiac Surgery, Ludwig Maximilian University of Munich, Munich, Germany; Department of Cardiac Surgery, Ludwig Maximilian University of Munich, Munich, Germany; Department of Cardiac Surgery, Ludwig Maximilian University of Munich, Munich, Germany; Munich Heart Alliance, Partner Site German Centre for Cardiovascular Disease (DZHK), Munich, Germany; Munich Heart Alliance, Partner Site German Centre for Cardiovascular Disease (DZHK), Munich, Germany; Department of Cardiac Surgery, University of Leipzig, Leipzig, Germany

**Keywords:** rapid-deployment prosthesis, aortic valve regurgitation, surgical aortic valve replacement

## Abstract

**OBJECTIVES:**

Surgical aortic valve implantation remains the therapeutic gold standard for aortic valve regurgitation. Due to annular dilatation and lack of calcification, transcatheter aortic valve replacement is not recommended. Although rapid deployment valves allow faster implantation and excellent haemodynamics, they are currently not recommended for patients with aortic valve regurgitation. This study retrospectively analysed the use of rapid deployment prostheses in patients with pure aortic valve regurgitation.

**METHODS:**

From 2014 to 2022, 444 rapid deployment valves were implanted. Since 2017 until 2022, 22 were used for patients with pure aortic valve regurgitation. This cohort was compared to 77 patients who had undergone rapid deployment valve implantation for pure aortic stenosis during the same time period. Both cohorts were analysed for major clinical outcomes, including pacemaker implantation, mortality, major adverse cardiovascular and cerebrovascular events (MACCE), and the need for redo surgery.

**RESULTS:**

In both groups, no valve intraoperative and postoperative valve revisions were required. Transvalvular gradients were comparable between the groups (ΔPmean/max 7.1/13.3 mmHg in the aortic valve regurgitation and 7.9/14.7 mmHg in the AS cohort), and there were no paravalvular leaks. The postoperative pacemaker implantation rate was 0% for the rapid deployment group and 1.3% for the conventional valve replacement group.

**CONCLUSIONS:**

These results suggest that rapid deployment valves can be safely applied for the treatment of patients with aortic valve regurgitation, even in the absence of calcification. This expands the surgeon’s armamentarium and can be especially useful in patients requiring extensive surgery where saving aortic cross-clamp time may be especially beneficial.

## Graphical Abstract

**Figure:**
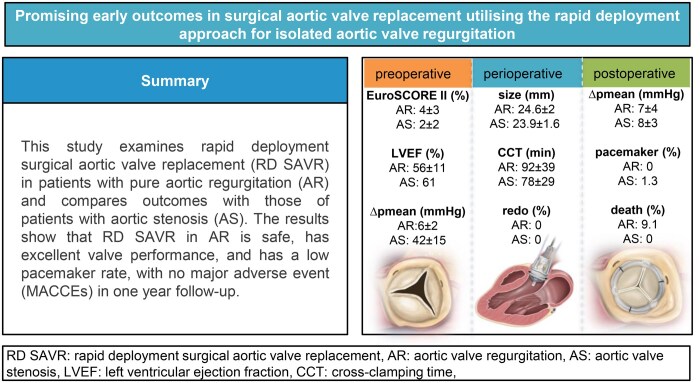

## INTRODUCTION

In recent years, there have been many developments in the treatment of aortic valve disease. Most of these are based on a reduction of procedure invasiveness, for example, by modified access route. Transcatheter aortic valve replacement (TAVR), providing the least invasive access route, has been integrated in all international treatment guidelines for the aortic valve stenosis in suitable anatomical conditions [1, 2]. However, for pure aortic valve regurgitation (AR) of native aortic valves, the use of TAVR prostheses is not yet recommended because annular calcification is critical for valve anchoring in TAVR [3]. For severe AR, as well as symptomatic patients with AR, surgical intervention (surgical aortic valve implantation [SAVR]) remains the therapeutic gold standard. There is some data on the Trilogy transcatheter heart valve (JenaValve Technology, Irvine, CA, USA) as an alternative to SAVR in patients with high-grade AR who have been classified as high risk by the heart team [2, 4].

New technological developments in surgical valve prostheses have led to the introduction of sutureless and rapid deployment (RD) valves, which simplify the surgical procedure and reduce operating times, especially cardiopulmonary bypass (CPB) and cross-clamping time. Recent literature describes a 39% reduction in CPB and a 48% reduction in aortic cross-clamping time [5]. Prolonged CPB and cross-clamp times are associated with increased morbidity and mortality, which is mainly due to a higher inflammatory response resulting in increased coagulopathies and renal insufficiency [6, 7].

Apart from increased mortality, morbidity and postoperative renal insufficiency, it is associated with prolonged ventilation times, low cardiac output and a higher need for blood transfusions [8].

Apart from its advantage in reduction of operative times the RD prosthesis furthermore provides excellent haemodynamic results, regardless of valve size. It is assumed that the replacement of the valve pledgets commonly used in conventional valve replacement, with the RD valve skirt which extends into the left ventricular outflow tract is responsible for reduced gradients [5, 9].

Although research has been carried out on RD valves in aortic valve stenosis and in combined pathologies, no previous study has adequately tested the effectiveness of RD SAVR in pure aortic regurgitation, which seems to be the reason for the current contraindication of RD SAVR in AR.

**Figure 1:**
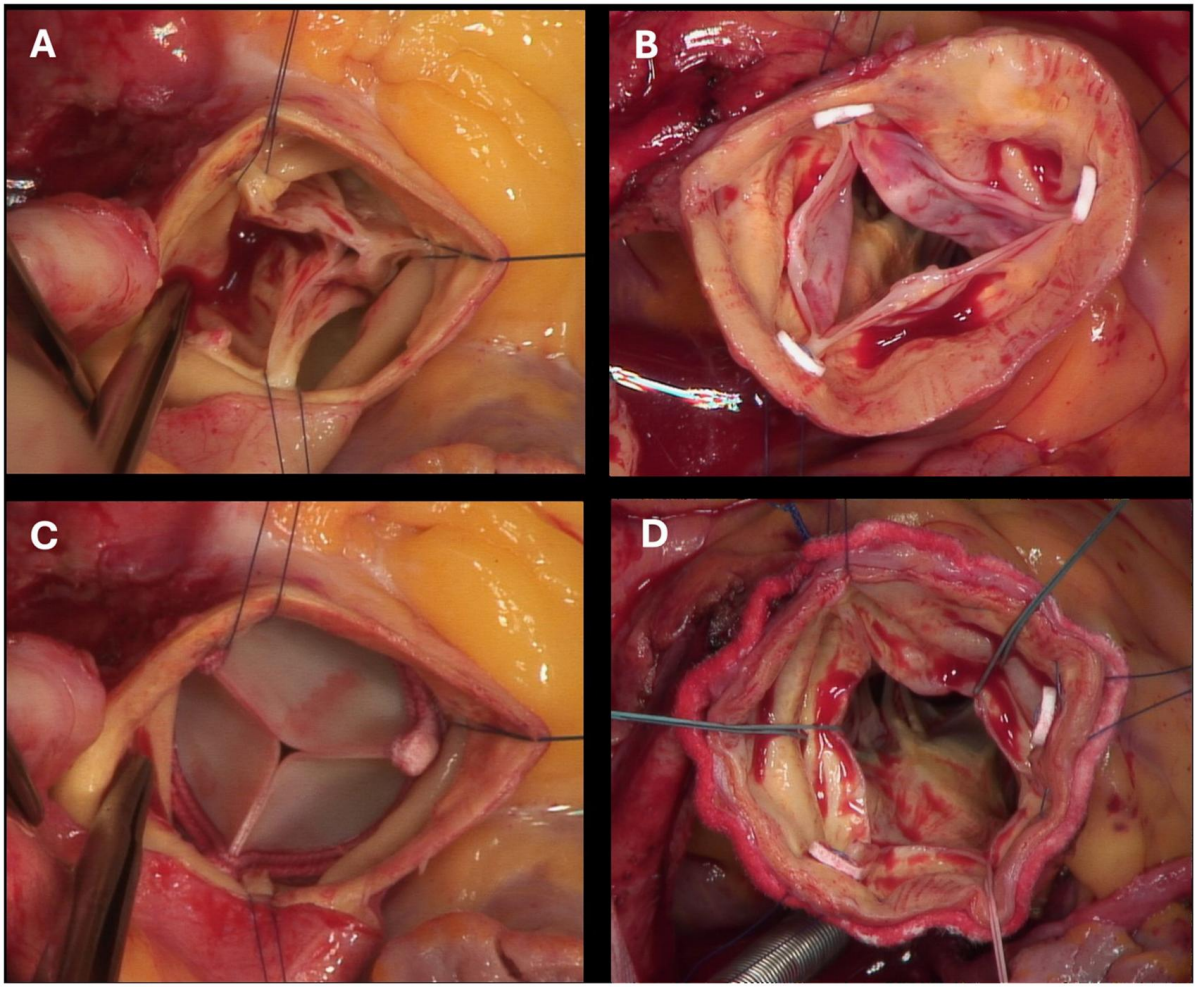
(**A**) View of aortic valve after incision of the aorta. Prolapsed pockets, no calcification. (**B**) View into the aortic valve after raising the valve at the commissures. Pockets thin, no calcification visible. LVOT without noticeable findings. (**C**) View into the LVOT after extraction of the pockets and attachment of the guiding sutures in the nadirs. Clean cut edges. (**D**) View of the implanted Intuity valve. Commissural sutures still *in situ*.

Below we present our experience with the use of RD SAVR for the treatment native aortic valves with pure aortic regurgitation (see Fig. 1).

## PATIENTS AND METHODS

We defined the study population as all patients who received RD SAVR for pure high-grade AR with a native aortic valve at our centre between 2014 and 2022. Since 2017, we started the RD SAVR approach in patients with pure AR. Those who received RD SAVR for pure aortic valve stenosis during the same period were chosen as the reference cohort. All patients received the EDWARDS INTUITY elite valve. Concomitant procedures were performed in both study collectives. Patient treated for bicuspid aortic valve disease were excluded. Written informed consent was obtained, and those who could not consent or were not contactable were omitted. Patients who underwent surgery fewer than a year before were also excluded, as this meant that a minimum of 1 year follow-up was not possible. A total of 22 patients with AR and 77 patients with AS (aortic valve stenosis) were included in the study.

Data collected as part of standard pre- and postoperative care in our centre were accessed. This data included demographic data, preoperative, intraoperative and postoperative data and a follow-up after 3 months and 1 year.

The data were recorded in patient management systems within the hospital. After data collection, the data were transferred REDCap electronic data capture tools hosted at the Department of Cardiac Surgery, LMU [10, 11]. The data were anonymized and then analysed using IBM SPSS version 25 (IBM Corp., Chicago, IL, USA).

A power analysis was performed beforehand with the program G*Power 3.1, based on an assumed effect size of Cohen’s d = 0.5, an alpha level of 0.05 and a power of 80%.

The sample size of 22 and 77 patients was calculated to be sufficient for the Chi-square test, as well as the t-test and Mann–Whitney U-test. For the Fisher’s exact test, the power was calculated at 60%, indicating a moderate detectability of the effect in categorical variables.

However, due to the smaller sample size, the power calculation should be considered an estimate, and caution is advised when interpreting the results due to the limited cohort.

To check for normal distribution, we used the Shapiro–Wilk test. For categorical variables, which are presented as a number and percentage, the *P*-value was calculated using either the Chi-square test or Fisher’s exact test. The Chi-square test was used if the expected frequency was at least 5, otherwise the Fisher’s exact test was used.

For continuous variables that are presented as mean ± standard deviation, the t-test for independent samples or the Mann–Whitney test was used. The t-test was used if the data followed a normal distribution (as determined by the Shapiro–Wilk test). When data did not follow a normal distribution, the Mann–Whitney U-test was used instead.

If the *P*-value was <0.05, the null hypothesis was rejected, and a significant difference was assumed.

The local ethics committee (Ethics Committee of the Ludwig-Maximilians-University of Munich, Institutional Review Board #20–474, date of acceptance December 8th 2020) reviewed and approved the study protocol including the establishment of a database (compliant with the WMA declaration of Taipei).

## RESULTS

Based on aortic valve pathology, we categorized the patients in two groups: pure AR as the cohort of investigation and pure AS, as the reference cohort. The average follow-up time was 13 ± 13 months and 16 ± 12 months, respectively, and the individuals were of comparable age at the time of surgery (73 ± 5 y AR vs 71 ± 7 y AS). Interestingly, more women underwent surgery for pure AR (54.5%), while more men did so for pure AS (64.9%).

In the AR group, the EuroSCORE II was higher with 4 ± 3% compared to 2 ± 2% in AS. The groups were comparable regarding classic cardiovascular risk factors, such as arterial hypertension, diabetes, hyperlipoproteinaemia and smoking, but differed regarding weight with patients in the AS group more likely being overweight (29 ± 5 AR vs 23 ± 10 kg/m^2^ AS). Eighteen percent of AR patients have been diagnosed with end stage renal disease with dialysis, in comparison to only 6.5% of AS patients, whereas 11.7% of the letter showed preoperative liver diseases, versus only 4.5% of the AR group. Previous cancer was also reported in 27.3% of the AR and only 16.9% of AS patients.

Both cohorts included patients who had already undergone cardiac surgery not involving the aortic valve. Two patients (9%) in the AR group had previously undergone preplacement of the ascending aorta, one patient in the AS cohort (1.3%) had previously undergone bypass surgery and one (1.3%) had undergone mitral valve surgery (see Table 1).

**Table 1:** Preoperative summary of patient characteristics and demographics

	All patients (*N* = 99)	AR (*n* = 22)	AS (*n* = 77)	*P* (AR vs AS)
Age (years)	71.0 ± 6.1	72.8 ± 5.2	71.3 ± 6.7	0.313^a^
Gender, *n* (%)				0.099^b^
Female	39 (39)	12 (54.5)	27 (35.1)	
Male	60 (60)	10 (45.5)	50 (64.9)	
meanEuroscore II (%)	1.8 ± 0.9	3.5 ± 2.5	1.9 ± 1.7	<.001^c^
Arterial hypertension, *n* (%)	88 (88)	21 (95.5)	67 (87)	0.447^d^
Diabetes mellitus, *n* (%)	26 (26)	5 (22.7)	21 (27.3)	0.601^b^
Smoking history, active, *n* (%)	9 (9)	2 (9.1)	7 (9.1)	>0.99^d^
Alcohol abuse, *n* (%)	6 (6)	1 (4.5)	5 (6.5)	>0.99^d^
Obesity (mean BMI), (kg/m^2^)	28.8 ± 5.2	23.0 ± 9.6	28.8 ± 5.0	0.019^a^
Hyperlipoproteinaemia, *n* (%)	68 (68)	13 (59.1)	55 (71.4)	0.308^b^
Family history, *n* (%)	16 (16)	3 (13.6)	13 (16.9)	>0.99^d^
Peripheral artery disease, *n* (%)	7 (7)	0 (0)	7 (9.1)	0.343^d^
Cerebral artery disease, *n* (%)	11 (11)	3 (13.6)	8 (10.4)	0.704^d^
Chronic lung disease, *n* (%)	13 (13)	2 (9.1)	11 (14.3)	0.728^d^
Cardiomyopathy, *n* (%)	2 (2)	0 (0)	2 (2.6)	>0.99^d^
Previous TIA, *n* (%)	2 (2)	0 (0)	2 (2.6)	>0.99^d^
Previous stroke, *n* (%)	2 (2)	0 (0)	2 (2.6)	>0.99^d^
End-stage renal disease with dialysis, *n* (%)	9 (9)	4 (18.2)	5 (6.5)	0.108^d^
Liver disease, *n* (%)	10 (10)	1 (4.5)	9 (11.7)	0.684^d^
Low cardiac output syndrome, *n* (%)	3 (3)	1 (4.5)	2 (2.6)	0.534^d^
Previous ICU, *n* (%)	1 (1)	1 (4.5)	0 (0)	0.214^d^
Immunocompromised, *n* (%)	4 (4)	2 (9.1)	2 (2.6)	0.187^d^
Previous cardiac surgery, *n* (%)	7 (7)	3 (14)	4 (5.2)	0.166^d^
CABG	1 (1)	0 (0)	1 (1.3)	>0.99^d^
MVR	1 (1)	0 (0)	1 (1.3)	>0.99^d^
Aortic replacement	2 (2)	2 (9.1)	0 (0)	0.048^d^
Previous cancer, *n* (%)	19 (19)	6 (27.3)	13 (16.9)	0.230^d^
New York Heart Association (NYHA), *n* (%)	70 (70)	13 (59.1)	57 (74)	0.096^b^
I	2 (2)	0 (0)	2 (3)	
II	23 (23)	2 (9.1)	21 (27)	
III	43 (43)	9 (40.9)	34 (44)	
IV	1 (1)	1 (4.5)	0 (0)	
Instable angina pectoris, *n* (%)	4 (4)	0 (0)	4 (5.2)	0.572^d^
Syncope, *n* (%)	9 (9)	2 (9.1)	7 (9.1)	>0.99^d^
CHA_2_DS_2_-VASc-score	3.6 ± 1.4	3.7 ± 1.1	3.5 ± 1.3	0.539^b^

Data presented as *n*(%) or mean ± standard deviation.

a t-test (for independent samples) for equality of means <0.05.

b Chi-square-test (Pearson).

c Mann–Whitney U-test.

d Fisher’s exact test.

The AR cohort showed a reduced LVEF (left ventricular ejection fraction) compared to the AS cohort (56 ± 11 AR vs 61 ± 10mmHg AS), although not statistically significant (*P *= 0.119, Mann–Whitney U-test). As expected, preoperative gradients were significantly higher in the AS cohort (see Table 2).

**Table 2:** Aortic valve specific data

	All patients (*N* = 99)	AR (*n* = 22)	AS (*n* = 77)	*P* (AR vs AS)
Left ventricular ejection fraction (mmHg)	61.7 ± 8.9	56.4 ± 11.1	60.7 ± 9.5	0.119^a^
Mean gradient (mmHg)	45.3 ± 14.0	5.7 ± 2.1	41.6 ± 15.3	<.001^b^
Maximal gradient (mmHg)	73.0 ± 19.5	10.6 ± 3.5	66.7 ± 23.2	<.001^b^
Aortic regurgitation, *n* (%)				
II/IV		3 (14)		
III/IV		7 (32)		
IV/IV		11 (50)		
Aortic orifice area, mean (cm^2^)			0.81 ± 0.25	
Endocarditis, *n* (%)	0 (0)	0 (0)	0 (0)	

Data presented as *n* (%) or mean ± standard deviation.

a Mann–Whitney U-test.

b t-test (for independent samples) for equality of means <0.05.

Partial sternotomy was performed in 30% of patients with AS and in 20% of the patients with AR.

Mean prosthesis size implanted in the AR cohort was 24.6 ± 2.1 mm, which corresponds roughly to the reference cohort with 23.9 ± 1.6 mm (*P *= 0.082 Mann–Whitney U-test). The 25 mm valve prosthesis was implanted most frequently in both groups. The 23 mm prosthesis was the second most frequently used in the AS group compared to the 27 mm prosthesis in the AR cohort.

As previously mentioned, concomitant procedures were performed in both populations. These differed significantly depending on the cohort; 61% of the patients in the AS group additionally received at least one bypass compared to 32% in the AR cohort. On the other hand, only 4% in the AS cohort had an additional aortic replacement, compared to 41% in the AR cohort. Only few patients underwent surgery of the mitral valve (9% AR and 6.5% AS), none on the tricuspid valve (see Table 3).

**Table 3:** Summary of valve specific perioperative data and relating the procedure

	All patients (*N* = 99)	AR (*n* = 22)	AS (*n* = 77)	P (AR vs AS)
Urgent, *n* (%)	3 (3)	2 (9.1)	1 (1.3)	0.123^a^
Partial sternotomy, *n* (%)	25 (25)	4 (18.2)	21 (27.3)	0.387^b^
Mean aortic prosthesis size (mm)	24.1 ± 1.7	24.6 ± 2.1	23.9 ± 1.6	0.082^c^
19, *n* (%)	1 (1)	0 (0)	1 (1)	
21, *n* (%)	11 (11)	3 (18)	8 (10)	
23, *n* (%)	34 (34)	5 (18)	29 (38)	
25, *n* (%)	40 (40)	7 (41)	33 (43)	
27, *n* (%)	12 (12)	7 (23)	5 (6)	
Concomitant procedures, *n* (%)				
Coronary artery bypass graft	54 (54)	7 (31.8)	47 (61.0)	0.015^b^
Mitral valve	7 (7)	2 (9.1)	5 (6.5)	0.650^a^
Tricuspid valve	0 (0)	0 (0)	0 (0)	
Aortic surgery	12 (12)	9 (40.9)	3 (3.9)	<.001^a^
Intraoperative data (min)				
Cardiopulmonary bypass time	111.5 ± 44.8	141.9 ± 53.4	113.8 ± 42.2	0.016^c^
Cross-clamp time	76.0 ± 29.6	92.1 ± 39.0	77.7 ± 28.7	0.102^c^

Data presented as *n* (%) or mean ± standard deviation.

a Fisher’s exact test.

b Chi-square-test (Pearson).

c Mann-Whitney U-test.

d Fisher's exact test.

A few patients received more than just one concomitant procedure. Specifically, in the AR cohort, patients underwent SAVR alone as frequently as SAVR with aortic replacement (32%), 18% underwent bypass surgery with SAVR, 9% underwent SAVR with aortic replacement and bypass, and 5% underwent SAVR with mitral valve surgery and bypass. Similarly, 5% had SAVR with mitral valve surgery. In the AS cohort, the distribution is different, most frequently SAVR with bypass was performed (56%), followed by SAVR alone (34%). Only one person underwent SAVR with aortic replacement (1%), but 3% underwent aortic replacement with bypass surgery. Equal numbers (3%) underwent SAVR with bypass and mitral valve surgery, slightly more (4%) underwent SAVR with mitral valve replacement (see Table 4).

**Table 4:** Subdivision into concomitant procedures per patient

Concomitant procedures, n (%)	AR (n = 22)	AS (n = 77)	P Ventilation time	P CPB	P CCT
Only SAVR	7 (32)	26 (34)	0.059	0.523	0.402
With CABG	4 (18)	43 (56)	0.237	0.689	0.607
With aortic replacement	7 (32)	1 (1)	0.614	0.122	0.127
With CABG and aorta	2 (9)	2 (3)	0.480	>0.99	>0.99
With mitral valve	1 (5)	3 (4)	0.637	0.655	0.655
With CABG and mitral valve	1 (5)	2 (3)	0.221	0.221	>0.99

Data presented as *n* (%) or mean ± standard deviation.

In line with the concomitant procedures, there is a significantly longer CPB (141.9 ± 53.4 AR vs 113.8 ± 42.2 min AS) and cross-clamping time (92.1 ± 39.0 AR vs 77.7 ± 28.7 min AS) in the main cohort, although no difference was found between the collectives when additionally analysed according to concomitant procedures (see Table 5).

**Table 5:** CPB and aortic cross-clamping time divided by concomitant procedure

	Cardiopulmonary bypass			Cross-clamping time		
Concomitant procedures, *n* (%)	AR	AS	*P*	AR	AS	*P*
Only SAVR	87.3 ± 17.9	81.8 ± 18.8	0.523	50.0 ± 10.6	54.6 ± 13.3	0.402
CABG	126.5 ± 13.6	125.5 ± 36.7	0.689	87. 8 ± 23.2	86.2 ± 24.2	0.607
Aortic replacement	187.6 ± 40.2	135.0 ± 0	0.122	124.4 ± 30.0	83 ± 0	0.127
CABG, aorta	211.0 ± 18.4	217.0 ± 66.5	>0.99	137.5 ± 9.2	142.5 ± 24.7	>0.99
Mitral valve	110. 0 ± 0	140 ± 64	0.655	81.0 ± 0	106.7 ± 46.2	0.655
CABG, mitral valve	160.0 ± 0	127.5 ± 24.7	0.221	100.0 ± 0	86.0 ± 22.6	>0.99

Data presented as *n* (%) or mean ± standard deviation.

Detailed transthoracic echocardiography was performed during inpatient stay, usually shortly before discharge. The gradients across the aortic valve prosthesis and LVEF were determined. A significantly worse LVEF was found in the AR cohort (51.8 ± 11.2% vs 60.7 ± 7.9% AS). A comparison of LVEF pre- and postoperatively also shows a significant reduction in the AR group only (*P *= 0.011, statistical test used: Wilcoxon signed rank test, Shapiro–Wilk).

In both groups, measured gradients were excellent and comparably low (ΔPmean 7.1 ± 3.5 AR vs 7.9 ± 3.2 mmHg AS, ΔPmax 13.3 ± 5.9 AR vs 14.7 ± 6.7 mmHg AS).

None of the patients experienced valvular complications during surgery, i.e. the valve did not have to be replaced or corrected for either deployment issues, instable anchoring or paravalvular leakage. Postsurgical complications were not significantly different.

One person per cohort (4.5% AR and 1.3% AS) developed new renal failure requiring dialysis postoperatively.

No person had to be reoperated due to prosthesis related issues, but 22.7% in the main cohort and 7.8% in the comparative group underwent rethoracotomy due to haemorrhage or tamponade.

The rate of postoperative pacemaker implantation is very low. In the AR group, 0 patients required pacemaker implantation postoperatively. In the AS group, one patient required pacemaker implantation postoperatively (*P = *0.582, Fisher’s exact test). The indication for implantation was high grade AV block.

No patient developed sepsis, SIRS (Systemic Inflammatory Response Syndrome) or endocarditis. On average, patients in both groups were discharged after 15 days.

We conducted a follow-up of all patients after (at least) 1 year. There were no further deaths, cardiac or otherwise, documented. No patient reported having experienced a myocardial infarction or stroke. Two patients in the AR cohort were hospitalized, one of whom had developed a bronchopulmonary infection and one of whom required thoracentesis. No individual underwent further cardiac surgery in the complete follow-up timeframe (see Table 6).

**Table 6:** Summary of collected data postoperatively, including complications and valve specific data as well as follow-up

	All patients (*N* = 99)	AR (*n* = 22)	AS (*n* = 77)	*P* (AR vs AS)
Left ventricular ejection fraction, [%]	61.7 ± 7.2	51.8 ± 11.2	60.7 ± 7.9	<0.001^a^
*P* pre- and postoperative	0.130	0.011	0.662	
Mean gradient (mmHg)	7.5 ± 3.2	7.1 ± 3.5	7.9 ± 3.2	0.381^b^
Maximal gradient (mmHg)	14.5 ± 6.9	13.3 ± 5.9	14.7 ± 6.7	0.583^a^
Intraoperative complications, *n* (%)				
Paravalvular leak, valve deployment (aortic valve)	0 (0)	0 (0)	0 (0)	
ECLS	0 (0)	0 (0)	0 (0)	
Postoperative complications, *n* (%)				
Reintubation	1 (1)	1 (4.5)	0 (0)	0.214^c^
Tracheotomy	1 (1)	1 (4.5)	0 (0)	0.214^c^
CPR	0 (0)	1 (4.5)	0 (0)	0.214^c^
Myocardial infarction	1 (1)	1 (4.5)	0 (0)	0.214^c^
Renal failure, permanent	2 (2)	1 (4.5)	1 (1.3)	0.384^c^
Major stroke/TIA	1 (1)	1 (4.5)	0 (0)	0.222^c^
Rethoracotomy (tamponade)	11 (11)	5 (22.7)	6 (7.8)	0.054^c^
New pacemaker implantation	1 (1)	0 (0)	1 (1.3)	0.582^c^
Paravalvular leak, dislocation of prothesis	0 (0)	0 (0)	0 (0)	
Redo surgery	0 (0)	0 (0)	0 (0)	
Postoperative sepsis, endocarditis	0 (0)	0 (0)	0 (0)	
Hospital and intensive care unit stay				
Ventilation time (h)	14.3 ± 6.3	15.7 ± 3.6	14.7 ± 6.7	0.035^a^
Intensive care unit stay (d)	3.1 ± 2.0	4.1 ± 2.5	3.1 ± 1.7	0.116^a^
Hospital stay (d)	13.5 ± 4.0	14.6 ± 6.7	14.7 ± 6.7	0.278^a^
Mortality in-hospital, *n* (%)	2 (2)	2 (9.1)	0 (0)	0.044^c^
1-year follow-up, *n* (%)				
Redo surgery, death, myocardial infarction, stroke, major bleeding	0 (0)	0 (0)	0 (0)	
Rehospitalization	2 (2)	2 (9)	0 (0)	

Data presented as *n* (%) or mean ± standard deviation.

a Mann–Whitney U-test.

b t-test (for independent samples) for equality of means <0.05.

c Fisher’s exact test.

## DISCUSSION

Interventions on the aortic valve have changed significantly in recent years as a result of the introduction of TAVR procedures, along with other developments. In cases of aortic valve stenosis, interventional valve replacement has now become established in older or previously diseased patients. At the same time, surgical replacement (SAVR) has moved towards more minimally invasive approaches.

Especially with the small, minimally invasive access route, the use of a RD prosthesis is of benefit as it facilitates implantation in the absence of a high number of sutures [12].

For pure aortic valve insufficiencies, however, the use of the TAVR method is not recommended as the TAVR valve cannot anchor in a non-calcified annulus. Other obstacles are usually the increase in elasticity of the tissue and often aortic dilatation.

Possible complications of such a procedure are embolization of the valve or valve malpositioning (TVEM). TVEM is, as described in the PANTHEON study of 2023, the most common complication of TAVR in AR and amounted to 12.4%. The most common cause of TVEM was malpositioning (32%), followed by oversizing (24%). An anchoring in the anulus could not be achieved in 20% of cases [13].

Nevertheless, some investigators have published their attempts of such off-label implantations [4, 14, 15].

Minimally invasive surgery combined with shorter intraoperative times is a suitable alternative for patients with AR with a higher preoperative risk or age, or those requiring a concomitant procedure, since prolonged intraoperative times are a risk for morbidity and mortality [16, 17].

There are no comparative studies that have used RD in AR. Nevertheless, in order to place our data in the context of existing literature, since our reference group is equivalent to this, we compared specific results with studies on RD SAVR in AS.

SURD-IR is a study that compared RD and sutureless valves in AS with concomitant procedures among others (22% bypass, mitral or tricuspid valve surgery 6%, aortic replacement 2%). In comparison, we recorded significantly higher average CPB (87.3 ± 38.5 min vs 141.9 ± 53.4 AR and 113.8 ± 42.2 min AS) and cross-clamping times (57.2 ± 28.7 min vs 92.1 ± 39.0 AR and 77.7 ± 28.7 min AS) with a very different distribution of concomitant procedures. When isolated SAVRs are compared, our cross-clamping time is similar (47.5 ± 22.9 vs 50.0 ± 10.6 min 'AR and 54.6 ± 13.3 min AS) [18].

The TRANSFORM study, which RD SAVR data reports a cross-clamping time of 49.3 ± 26.9 min and CPB of 69.2 ± 34.7 min, corresponded with our values. We also recorded similar values for pure SAVR as the GARY-Study, they report 50 (41–69) min for cross-clamping and 81 (64–103) min for CPB [19, 20].

Since Edwards’ RD prosthesis is currently only officially approved for aortic valve stenosis and combined pathologies, its use for pure regurgitation is off label. Accordingly, there are only isolated case reports in the literature of individuals who have received an RD prosthesis for pure AR, as well as some data on the use of sutureless prostheses in the AR setting [21, 22].

After using the Intuity valve increasingly more often at our hospital since 2014 (a total of 444 deployments during the observation period), we expanded the range of applications to include pure insufficiencies. No intraoperative valvular issues were detected in any of the AR and AS patients, neither during deployment of the valve nor paravalvular leaks after implantation. No PVL (paravalvular leak) was seen at the end of the inpatient stay either. No patient required a valvular redo surgery. We measured excellent haemodynamic gradients across the valve before discharge in both groups (ΔPmean 7.1 ± 3.5 AR and 7.9 ± 3.2 mmHg AS). These gradients are lower than in comparative studies: the GARY study reported a mean gradient of 10.0 ± 4.3 mmHg, SURD-IR recorded 11.3 ± 4.9 mmHg and the TRANSFORM study a mean gradient of 10.3 ± 3.8 mmHg, albeit the latter after 1 year.

With AR in particular, it is important that the valve is not oversized ‘for safety reasons’, as otherwise a pacemaker is often required postoperatively. Comparative literature describes a pacemaker implantation rate for RD SAVR in AS of 8.4% by SURD-IR, 8.8% in the GARY study and 12.3% in the TRANSFORM study, which is significantly higher compared to conventional implantation technique [19, 20, 23]. In our AS cohort, we report a significantly lower implantation rate (1.3%), and in the AR cohort, there was not a single patient who required a pacemaker.

Both GARY and the SURD-IR registry showed significantly higher stroke rates with the use of RD prostheses in contrast to conventional valves. GARY recorded 2.2%, TRANSFORM of 2.6% and SURD-IR even 2.8% [19, 20, 23].

In our overall cohort, stroke rate is significantly lower, at only 1%, but it is 4.5% in the AR cohort. However, this patient was admitted for emergency surgery for Stanford type A dissection and already presented preoperatively with a reduced LVEF of 40% and a EuroScore II of 9.3%. No stroke has been documented in patients undergoing elective surgery for aortic regurgitation.

Compared to the SURD-IR registry in patients with concomitant procedures, we recorded a higher mortality rate (1.4–3.5% vs 4.5% in non-urgent AR patients) [18].

There were no deaths in the reference cohort. The high mortality rate in the AR cohort can be explained by the fact that the sample is so small and that the patient treated as an emergency who initially had a higher expected mortality strongly affects the overall cohort mortality. Inclusion bias may furthermore affect the study results in this respect.

The length of stay in the ICU and in hospital is comparable in both groups (4.1 ± 2.5AR and 3.1 ± 1.7 d AS, 14.6 ± 6.7 AR and 14.7 ± 6.7 d AS).

Interestingly, the ICU length of stay is longer than reported in the SURD-IR registry (median 1(1–3)), but comparable to GARY (4 ± 7 d), which could be due to a fast track concept adopted at some hospitals with extubation in the operating room. The mean hospital stay is again comparable to GARY (13 ± 10 d), and slightly longer than SURD-IR (median 9(7–14)).

In our 1-year follow-up, we recorded no major adverse cardiac and cerebrovascular events (MACCEs).

### Limitation

Due to the small sample size in this single-centre study, the generalizability of the results to larger patient groups is limited. The frequent use of the Intuity prosthesis in our clinic could affect reproducibility in centres less familiar with the procedure.

A power analysis confirmed sufficient statistical power (>0.8) for the Chi-square test, the t-test and the Mann–Whitney U-test, but the Fisher test only showed a power of 60%, indicating moderate detectability of the effect. As this is a retrospective study, there may be selection bias due to patient consent.

Randomization was not possible as the choice of prosthesis depended on the surgeon. The follow-up period of 1 year allows only limited conclusions about the long-term performance and durability of the valves, and confounding factors such as concomitant procedures and high-risk patients may influence the MACCE results. A longer observation period and, if necessary, a larger cohort are needed to generalize our findings to other centres.

In the present cohort, the AR is not attributable to a diagnosed Marfan syndrome or rheumatic diseases. Accordingly, further data collection and studies are needed to be able to make statements about RDSAVR in various aetiologies of AR.

## CONCLUSION

In conclusion, we believe that the use of RD prosthesis in the AR setting is a safe method. We recorded no valvular complications and a significantly lower pacing rate than documented in previous studies. We also recorded excellent gradients during follow-up. Investigation of a larger cohort allowing subgroup analyses of elective patients as well as patients with specific concomitant procedures could further elucidate the role of RD valves in the treatment of patients with pure AR so that the list of indications can be expanded.

## Data Availability

The data that support the results of this study are available on request from the corresponding author.

## ABBREVIATIONS

AR: Aortic valve regurgitation
CPB: Cardiopulmonary bypass
RD: Rapid deployment
SAVR: Surgical aortic valve implantation
TAVR: Transcatheter aortic valve replacement
